# Potential environmental impact of mycelium composites on African communities

**DOI:** 10.1038/s41598-024-62561-7

**Published:** 2024-05-24

**Authors:** Stefania Akromah, Neha Chandarana, Jemma L. Rowlandson, Stephen J. Eichhorn

**Affiliations:** Bristol Composites Institute, School of Civil, Aerospace, and Design Engineering, Faculty of Science and Engineering, University of Bristol, University Walk, Bristol, BS8 1TR UK

**Keywords:** Life cycle assessment, Sustainable solutions, Building materials, Green building, Building and construction, Sustainable development goals, Composites, Environmental impact

## Abstract

The ecological and economic benefits of mycelium composites offer a promising opportunity for supporting sustainable development in Africa. This study focuses on assessing the environmental impact of mycelium composites for building and construction (MCBs) by conducting a life cycle assessment (LCA) in the context of Africa. It is demonstrated that the potential environmental impact of MCBs is substantially influenced by the use and source of electrical power for autoclaves, incubators, and ovens, making the culturing and post-processing phases the major environmental hotspots. The impact of MCB production is also relative to the energy mix of specific countries, being higher in countries that rely on fossil fuel energy (e.g., South Africa) and lower in those that rely more on renewable sources (e.g., Democratic Republic of the Congo, DRC). Furthermore, the impact of MCB production is found to be sensitive to travel distance, suggesting that situating production facilities closer to agricultural, agro-industrial, and/or forestry waste sources could be more beneficial than interregional sourcing, for example. It is also demonstrated that MCBs have the potential to be a more ecologically sustainable alternative to some conventional construction materials (e.g., concrete) over an entire life cycle. Based on the insights obtained from this LCA, some recommendations have been proposed to address potential environmental repercussions pre-emptively and proactively: this is particularly important for nations, mainly in the Global South, that exhibit low resilience to climate change due to limited economic resources. Furthermore, with the rapid expansion of mycelium composite technology, there is a need to increase awareness about its potential environmental impact and, ultimately, to mitigate its potential contribution to pressing environmental concerns (e.g., global warming and climate change). Consequently, this study also adds to the existing body of literature on LCA studies, delineating key factors for consideration in future LCA studies and providing guidance for the sustainable establishment and expansion of this technology.

## Introduction

Mycelium composites (MCs) are a class of materials based on organic particles bound in a network of fungal filaments (known as mycelium)^1^. The nature of these materials renders them biocompatible, biodegradable, recyclable, and compostable^2^. The organic substrate is often sourced from agricultural, agro-industrial, and forestry residues, thus, adding more value to materials that would otherwise be considered waste^3^. This also presents an alternative for managing organic waste, with the potential to mitigate the threats to the environment associated with emissions (e.g., carbon dioxide (CO_2_) and methane (CH_4_))^4,5^ generated during conventional waste management practices, e.g., landfilling^6–9^. In addition to the ecological benefits, mycelium composite production is considered cost- and energy-efficient due to the sourcing of raw materials and the minimal requirement for high-end complex manufacturing processes^1^.

In a previous publication^10^, we proposed and reviewed mycelium composites as a potential solution to environmental and socio-economic challenges in Africa, capitalizing on the substantial biomass reserve generated by the agricultural, agro-industrial, and forestry sectors. In Ghana, for example, the annual post-harvest waste (excluding agro-industrial, forestry, and consumer waste) amounts to approximately 30 Mt, of which 70% is composed of yam, cassava, plantain, and cocoa residues^6^. It is also estimated that an average of ~ 16% by weight (equivalent to an estimated value of $4 billion)^11^ of the continent’s agricultural produce is lost as waste during storage, processing, and packaging operations^12–14^. This waste is typically abandoned in the open field, incinerated, or landfilled, threatening the health of the ecosystems^6,7^. As a potential solution, mycelium composites, which are bio-based materials made using organic waste, can be used to repurpose this biomass into valuable products. Thus, holding the potential to mitigate the impact associated with solid organic waste while reducing the overall carbon footprint of industries (e.g., building and construction) that traditionally rely on fossil-fuel derived materials. This is corroborated by recent life-cycle assessments (LCAs), conducted by Stelzer et al.^15^ and Livne et al.^16^, showing that mycelium composites can act as a CO_2_ sink and that a unit brick of mycelium has a lower environmental footprint than a unit block of concrete, for example.

This study aims to assess the potential environmental impact of mycelium composites for building and construction (MCBs) in Africa, using Ghana as a case study. In this work, cradle-to-gate and cradle-to-grave LCA studies were conducted to identify the potential environmental hotspots of MCB production on a small industrial scale and to assess the potential environmental impact of MCBs over their entire life cycle, with a comparison made to concrete bricks. Furthermore, recommendations have been proposed to address some of the identified environmental repercussions pre-emptively and proactively: this is particularly crucial for countries that may face challenges in addressing catastrophic environmental degradation, after it occurs, due to financial constraints. The findings of this study can be adopted as a guide for developing sustainable practices in the production, application, and disposal of MCs, not only within the context of building and construction but also across a broader range of applications (e.g., packaging).

## Methodology

This LCA study is based on ISO 14040^17^ and ISO 14044^18^ standards. The goal and scope, and inventory are defined in the following sections.

### Goal and scope definition

The goal of this study is to identify the potential environmental hotspots of MCB production, and to assess the full life-cycle impact of MCBs compared to traditional concrete bricks using both cradle-to-gate and cradle-to-grave LCA methods. This work is contextualised within Africa using Ghana as the primary case study and extending its scope through a comparative analysis with other countries like the Democratic Republic of the Congo (DRC), Ethiopia, Kenya, Zambia, Mozambique, and South Africa. For Ghana, this model assumes that the MCB production facility is located near the Crop Research Institute of the Council of Scientific & Industrial Research (CSIR) in the Ejura-Sekyedumase district (Ashanti region), one of the major agricultural hubs of the country^19^. This location provides both an abundant biomass reserve as well as scientific research support, which, as suggested in our previous review^10^, can be valuable for the establishment and expansion of the MC technology in Africa.

**Figure 1:**
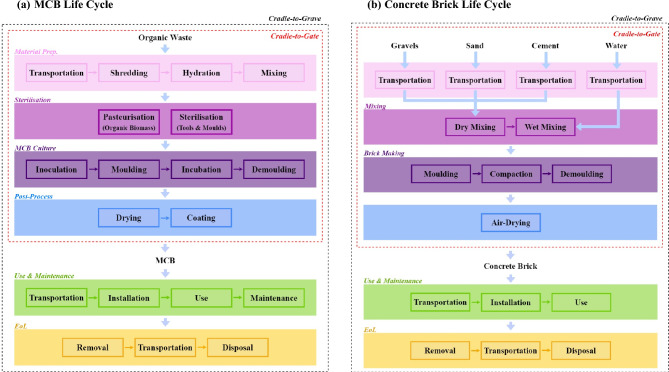
Flowchart of mycelium composite production showing the system boundaries for the cradle-to-gate and cradle-to-grave LCAs. (**a**) MCB life cycle. (**b**) Concrete brick life cycle.

The MCB model proposed in this study is based on information found in the literature^1,3,20^. Fig. 1a summarises the production chain for MCBs within the system boundaries defined in this LCA study. In summary:

1. Biomass is sourced from agricultural, agro-industrial, and forestry waste, and sterilised to prevent microbial contamination and maximise fungal growth^21^.
2. A grain spawn is prepared using biomass with high nutritional value, typically composed of glucose (e.g., rye berries) or starch (e.g., corn kernels)^22^.
3. Biomass with low nutritional value (typically composed of lignocellulose) is used as the bulk substrate; it is inoculated with the grain spawn and incubated, using the single or double-incubation methods^23^, to obtain as-grown MCs^24^.
4. Finally, the as-grown MCs are removed from the moulds and dried to cease biological activities and obtain the final MC products^25^ (e.g., MCBs).

The chosen functional unit is based on production volume, i.e., a cubic meter of solid bricks (no core holes); hence, the material and energy requirements are modelled for 1 m^3^ of MCBs and 1 m^3^ of concrete bricks, respectively. This decision was informed by the notable difference in the densities of the two materials (i.e., ρ_MCB_ = 300 kg m^−3^ and ρ_concrete_ = 1900 kg m^−3^)^3,26^, making mass a less suitable functional unit in this LCA model which assumes a 1:1 concrete-to-MCB replacement ratio. We acknowledge that a 1:1 ratio may prove impractical for the proposed application, considering the substantial disparity between the physical and mechanical properties of MCBs and concrete bricks. In fact, the reported compressive strength of mycelium composites can be as low as 0.17 MPa for wheat straw substrates^27^, while the strength of concrete typically exceeds 17 MPa^28^. These disparities limit the applicability of the findings to applications that require a minimal load bearing capacity where MCBs and concrete bricks can be employed interchangeably (e.g., partitioning walls in residential buildings). As suggested by the literature, mycelium composites are currently far from being suitable for semi-load bearing applications^3^. In future studies, as research in this field progresses and mycelium composites find broader applications (in structural contexts), the choice of functional unit may need to be reevaluated to accommodate increased compressive strength requirements. Figure 1b illustrates a summary of the concrete brick production process as would be manufactured in Ghana and several other African countries^29^. Typically: three parts of gravel, two of sand, and one of Ordinary Portland Cement (OPC) are combined and water (in a ratio of 0.5 to cement) is then added to the dry mix. The concrete slurry is poured into moulds, pressed using a manual compactor, and air-dried to obtain concrete bricks. This process is usually labour intensive and does not rely on any advanced equipment.

A combination of methods employed in recent LCA studies^15,30,31^ on MCBs was used to provide a comprehensive impact assessment covering the potentials for global warming, acidification, photochemical ozone creation, eutrophication, and water scarcity arising from the production, use, and disposal of MCBs. The ReCiPe method (V1.1, 2016)^32^ was used to assess endpoint damages to human health, ecosystems, and resources. The endpoint damages are calculated by normalising and weighting midpoint damages (e.g., global warming, ionising radiation, and ecotoxicity); scores are then assigned to each weighted value and summed into single scores^33^. The Intergovernmental Panel on Climate Change’ Global Warming Potential method (IPCC GWP100; V1.0, 2021)^34^ was used to assess the global warming potentials in kilograms of CO_2_ equivalent (kgCO_2_-eq.). The methodology developed by the Centre of Environmental Sciences at Leiden University (CML; V2.05, 2001)^35^ was used to assess potentials for acidification in kilograms of sulphur dioxide equivalent (kgSO_2_-eq.); eutrophication in kilograms of phosphate compounds equivalent (kgPO_4_-eq.); and photochemical ozone creation in kilograms of nitrogen compounds equivalent (kgNOx-eq.). The water scarcity-related impacts were assessed using the Available Water Remaining method (AWARE; V1.04, 2021)^36^.

### Life cycle inventory

The LCA inventory data was obtained from the literature and the EcoInvent 3 (2021) database^37^ in the SimaPro software (version 9.3.0.3; https://simapro.com/)^38^. Table 1 summarises the assumptions made in this study, while Table 2 presents the selected EcoInvent datasets for each input parameter. Global average datasets (GLO and RoW) were used for all parameters, except for “electricity”, as specific datasets for Ghana (GH) were unavailable. We acknowledge that global average datasets may not accurately represent the material/energy flows and resulting emissions for a specific geographical area^37^ (such as Ghana). Hence, future work should focus on defining the degree of uncertainty of this proposed model and, if possible, developing more representative models.

**Table 1:** List of assumptions made to calculate the input parameters for the cradle-to-gate and cradle-to-grave LCA models.

Parameter	Mycelium composite bricks (MCBs)	Concrete bricks
Density	300 kg m^−1^, i.e., max. reported density for MCs^3^	1900 kg m^−1^ ^26^
Composition	Grain spawn: 20 wt% corn kernels Substrate: 80 wt% coconut coir Mycelium: excluded from LCA (Biomass composition based on^3^)	Gravel: 3 parts Sand: 2 parts Cement: 1 part Water (0.5 of cement) (Composition based on^29^)
Water	Biomass hydration water: Lost by evaporation when drying Wastewater from brick making: Contaminated with organic matter from biomass	Hydration water: Retained in the brick as bound water Wastewater from brick making: Contaminated with gravel/sand/cement
Brick mass	Equal to the sum of the mass of corn kernel and coconut coir Remains constant throughout production process	Equal to the sum of the mass of gravel, sand, cement, and water Remains constant throughout production process
Equipment	Water, electricity, and fuel calculated from: UNTHA™ shredder^43^ Bubba’s Barrels™ substrate steamer^44^ Synchem™ autoclave^45^ Cleanroom (Cleanroomshop™)^46^ Grow tent (Gorilla Grow Tent™)^47^ Shanghai Jimei Industry™ industrial oven^48^	Manual concrete brick making machine; process is labour intensive
Electricity consumption (breakdown)	Shredder: 3.1 kWh Steamer: 50.4 kWh Autoclave: 180 kWh Cleanroom (filter fans + LED lights): 676.8 kWh Grow tent (filter fans + LED lights): 570.6 kWh Electric oven: 1632.0 kWh	None
Travel distance	Sourcing to production facility: 3.5 km Production facility to user: 3.5 km User to disposal facility: 3.5 km	Same as MCBs
Mode of transportation	Biomass: Sinotruck HOWO™ light cargo truck^49^ MCBs to user: Sinotruck HOMO™ cargo truck^50^ Maintenance: Passenger car (unspecified) Waste to disposal site: Sinotruck HOMO™ cargo truck^50^	Gravel and sand: HH Engitech™ tipper truck^51^ Bricks to user: DAF™ flatbed truck^52^ Waste to disposal site: Sinotruck HOMO™ cargo truck^50^
Auxiliaries	Disposable: Polypropylene (PP) grow bags Nitrile gloves Reusable: Mixing bins Moulds Others (e.g., scalpels, forceps, etc.)	Disposable: None Reusable: Moulds Others (e.g., pans, shovels, wheelbarrows, etc.) (Inventory from conventional production process^29^)
Production timelines	Estimated total time: ~ 8 weeks^39^ Material preparation and sterilisation: ~ 2 days Culturing: ~ 7 weeks Post-processing: ~ 5 days	Estimated total time: ~ 1 week^29^ Brick making: ~ 1 day Drying: ~ 6 days
Use and maintenance	Moisture/insect proof surface coating: TERM-Seal™ sealant, primer, and repellent^53^ Regular maintenance every two years^54,55^	No major maintenance over the considered timescales
Lifespan	20 years, i.e., estimated lifespan of MCs^54,55^	100 years, i.e., estimated lifespan for concrete bricks^56^
Waste management	Landfilled includes: Biomass waste (i.e., 10% surplus) Disposables (gloves, PP bags) Waste MCB (EOL)	Landfilled includes: Waste gravel/sand/cement (i.e., 10% surplus) Waste concrete bricks (EOL)

**Table 2:** Materials and energy inputs (including 10% surplus) and outputs for 300 kg of MBCs and respective EcoInvent 3 datasets, accessed through SimaPro (v9.3.0.3)^38^.

Inputs	Amount	Unit	SimaPro dataset
Nutrient	66.0	kg	Maize grain {RoW}|market for maize grain|Cut-off, U
Substrate	264.0	kg	Coconut husk {GLO}|market for coconut husk|Cut-off, U
Varnish	0.03	kg	Acrylic varnish, without water, in 87.5% solution state {RoW}|market for acrylic varnish, without water, in 87.5% solution state|Cut-off, U
PP grow bags	0.11	kg	Polypropylene, granulate {GLO}|market for|Cut-off, U
Isopropanol	0.9	kg	Isopropanol {RoW}|market for isopropanol|Cut-off, U
Nitrile gloves	0.29	kg	Acrylonitrile–butadiene–styrene copolymer {GLO}|market for|Cut-off, U
Transport	3860.0	kg km	Transport, freight, light commercial vehicle {RoW}|market for transport, freight, light commercial vehicle|Cut-off, U
Water (total)	4250.0	kg	Tap water {RoW}|market for|Cut-off, U
Electricity	3272.9	kWh	Electricity, medium voltage {GH}|market for electricity, medium voltage|Cut-off, U

Outputs	Amounts	Unit	SimaPro Dataset
MCB	300.0	kg	Biopolymer
Waste biomass	30.0	kg	Biowaste {RoW}|treatment of biowaste, open dump|Cut-off, U
Wastewater	4.25	m^3^	Wastewater, average {RoW}|market for wastewater, average|Cut-off, U
Waste PP bags	0.11	kg	Waste polypropylene {RoW}|treatment of waste polypropylene, sanitary landfill|Cut-off, U
Waste gloves	0.29	kg	Waste plastic, mixture {RoW}|treatment of waste plastic, mixture, sanitary landfill|Cut-off, U

Within the scope of this LCA, an MCB unit consists of 20 wt% maize grains (nutrient-rich biomass) and 80 wt.% coconut coir (bulk substrate). For simplification, the mycelium component is omitted, and we assume that the mass of an MCB remains constant throughout the production process since there is no fungal activity. In reality, due to fungal activity, the mass of the substrate fluctuates due to loss of substrate and metabolic conversion of biomass to CO_2_ and H_2_O^39^. The total manufacturing timeline is estimated to be approximately 8 weeks^39^:  ~ 2 days for material preparation, 7 weeks for incubation, and ~ 5 days for drying. The material and energy requirements listed in Table 2 were calculated based on the average consumptions for various commercial equipment and vehicles of which some examples are provided. It is assumed that mixing bins, moulds, and other auxiliaries (e.g., scalpels, forceps, scissors, etc.) are reusable and sterilised by an autoclave, while gloves and polypropylene (PP) grow bags are disposed of after each single use. To account for production waste, a 10% surplus (which falls within the acceptable scrap rate range)^40,41^ was added to all input parameters. Production and post-consumer solid waste is landfilled, although, in real-life scenarios, biomass and MCBs can be composted or recycled^31,42^. Wastewater, including water from the substrate steamer, the autoclave, production wastewater, and hydration water, is assumed to be contaminated with organic matter from the biomass and discharged.

## Results and discussion

### Cradle-to-gate LCA for MCB production

**Figure 2:**
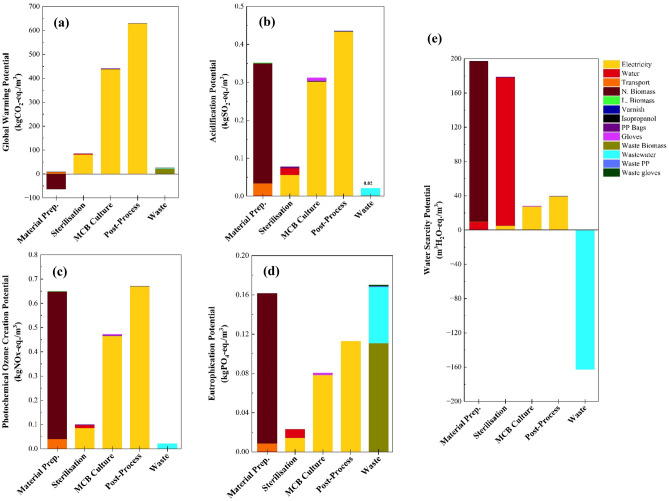
Environmental impact of MCB production under the impact categories of (**a**) global warming potential (GWP), (**b**) acidification potential (AP), (**c**) photochemical ozone creation (POCP), (**d**) eutrophication potential (EP), and (**e**) water scarcity potential (WSP).

Figure 2 shows the potential environmental impact of production under the impact categories of global warming (GWP), acidification (AP), photochemical ozone creation (POCP), eutrophication (EP), and water scarcity (WSP) per cubic meter of bricks. A summary of these results is also provided in Tables S1 and S2 in ‘Supplementary Material’.

The post-process phase shows the highest GWP (629.7 kgCO_2_-eq., i.e., 55.8%), AP (0.4 kgSO_2_-eq., 37.8%), and POCP (0.7 kgNOx-eq., 0.4%). The culturing phase ranks second in the context of GWP with 441.7 kgCO_2_-eq. (39.1%); it also has a significant contribution on AP (25.9%) and POCP (25.6%). As suggested by Fig. 2 (Figs. S1, S2, and S3), electricity consumption is the key factor influencing the impact of these processes (i.e., MCB culturing and post-processing) in all assessed categories. The high impact associated with these energy intensive processes is closely related to the energy source and, hence, the electricity mixes of specific countries (later discussed in Section "Sensitivity analysis of MCB production"). Since non-renewable energy sources (e.g., coal) are more detrimental to the environment as compared with renewable ones^57^, the environmental impact of MCB production is expected to be greater in countries that rely primarily on fossil fuels (e.g., South Africa). Thus, the results presented in this section are only specific to Ghana where ~ 61.0% of the total electrical power is generated from thermal energy from fossil fuels, ~ 38% from hydropower and ~ 1% from other renewable energy sources^58,59^.

The material preparation phase which covers sourcing, transportation, and hydration of agricultural waste, accounts for the highest WSP, contributing to 44.5% (197.2 m^3^ H_2_O-eq.) of the total excluding wastewater. It also makes a significant contribution to EP (0.2 kgPO_4_-eq., i.e., 29.1%), POCP (0.7 kgNOx-eq., i.e., 34.0%), and AP (0.4 kgSO_2_-eq., i.e., 29.2%). This contribution can be attributed to the nutrients and water required to grow nutrient-rich biomass^31^ like maize. Nutrients are supplemented by fertilisers with high concentrations of phosphorus and nitrogen compounds^60^. Meanwhile, the GWP of the material preparation phase was found to be negative due to the carbon uptake capacity of biotic materials^61^.

Waste accounts for 31.0% of the total EP (0.55 kgPO_4_-eq.) due to its high organic composition consisting of waste biomass and biomass-contaminated wastewater (as previously explained, biomass generally has a high EP). However, waste has a negative WSP (− 162.53 m^3^ H_2_O-eq.) with wastewater, typically discharged back into the environment^62^, being the main contributor.

### Sensitivity analysis of MCB production

A sensitivity analysis was conducted to assess the influence of electricity consumption, water usage, and amount of biomass on the environmental impact of MCB production when the respective input values (previously listed in Table 2) are changed by ± 100%. The chosen percentage variation serves to evaluate the sensitivity of the system to the parameters under two distinct scenarios: (1) in the absence of the specified parameter (i.e., lower bound limit, L = − 100%); (2) when the amount of electricity, water, or biomass is twice the amount estimated in the LCA model (i.e., upper bound limit, H =  + 100%), for example, if the initial input values (in Table 2) were underestimated. The sensitivity analysis also covers sensitivity to energy mix (country specific) and travel distance. A further analysis was conducted, exploring the potential for use of alternative fuels that are considered more affordable, reliable, and accessible compared to electricity^63^. The term “baseline” used henceforth refers to the impact values obtained from the initial LCA model (Section "Cradle-to-gate LCA for MCB production").

#### Electricity, water, and biomass consumption

**Figure 3:**
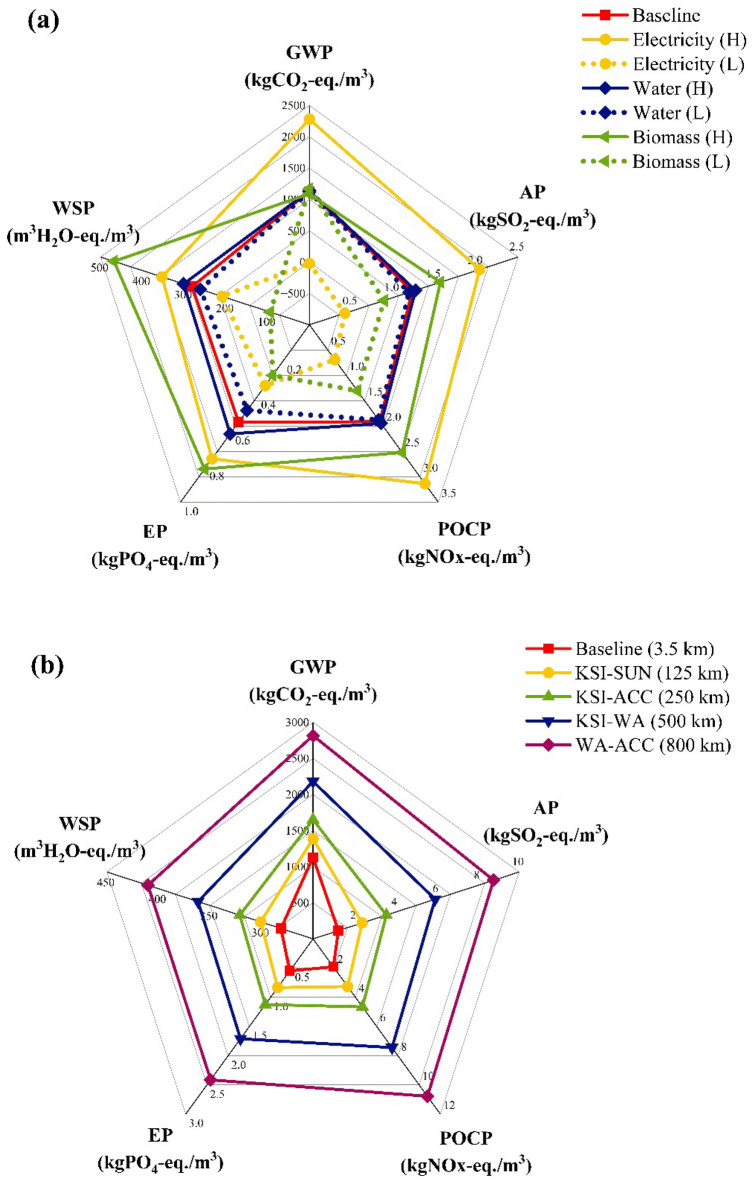

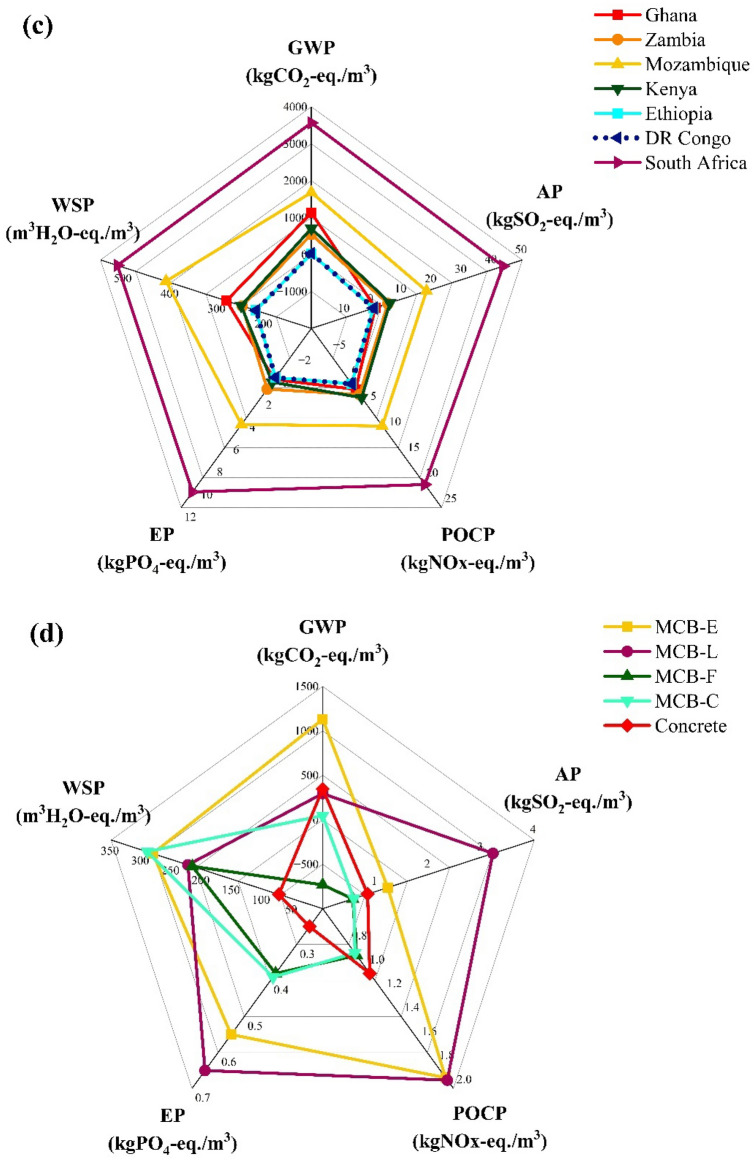
Sensitivity equivalent emissions from MCB production to changes in (**a**) electricity, water, and biomass (H = high, i.e., + 100%; L = low, i.e., − 100%); (**b**) travel distance; (**c**) energy mix; (**d**) fuel source.

As evident in the results presented in Fig. 2, and in agreement with the LCA study conducted by Stelzer et al.^15^, electricity, water, and biomass contribute significantly to the overall environmental impact of MCB production. Figure 3a (data provided in Table S3) shows the sensitivity of the equivalent emissions from MCB production to electricity, water, and biomass variations. As shown, electricity has the highest influence on GWP, AP, and POCP due to its substantial usage throughout the manufacturing process and the larger magnitude of its impact on the environment, as highlighted in the previous results. Specifically, a ± 100% change in electricity affects GWP by ± 101.8% of the baseline kgCO_2_-eq.; AP by ± 65.6% of the baseline kgSO_2_-eq.; and POCP by ± 64.0% of the baseline kgNOx-eq. Electricity also has a significant influence on EP, with the kgPO_4_-eq. changing by ± 37.5%, and on WSP, with the m^3^ H_2_O-eq. changing by ± 25.6%. However, EP and WSP show a higher sensitivity to the weight of biomass (as larger quantities require more fertiliser and water), with the EP changing by ± 48.1% of the baseline kgPO_4_-eq. and the WSP changing by ± 66.6% of the baseline m^3^H_2_O-eq. AP and POCP are also sensitive to variations in biomass weight with AP changing by ± 27.4% of the baseline kgSO_2_-eq. and POCP changing by ± 31.8% of the kgNOx-eq.

#### Travel distance

While the baseline LCA study assumes that agricultural waste is sourced from local farmers within the same community, it is important to note that this may not always be the case. For example, biomass may be sourced from regions with higher agricultural waste yields or from those producing specific biomass compositions required for MCBs with specific desired properties. For this reason, the distances between four major regional capitals in Ghana were used to assess the sensitivity of emissions generated from MCB manufacturing to travel distance: Kumasi-Sunyani (125 km); Kumasi-Accra (250 km); Kumasi-Wa (500 km); Wa-Accra (800 km). Figure 3b (results in Table S4) illustrates the relationship between travel distance and the emission equivalents for GWP, AP, POCP, EP, and WSP, showing a linear correlation. The slope of each line represents the corresponding equivalent emission per kilometre, i.e.: 2.12 kgCO_2_-eq. for GWP; 9.5 × 10^–3^ kgSO_2_-eq. for AP; 1.1 × 10^–2^ kgNOx-eq. for POCP; 2.3 × 10^–3^ kgPO_4_-eq. for EP; 1.6 × 10^–1^ m^3^H_2_O-eq. for WSP. It is important to note that these are approximations, and they may overestimate or underestimate the actual emission equivalents per kilometre which, with regards to travel distance, are sensitive to various factors: type and quality of fuel; age and efficiency of vehicles used; state of the Ghanaian transportation infrastructure, etc.^64^.

#### Energy mix

Figure 3c (data in Table S5) presents the sensitivity of MCB production to the energy mix of specific countries. Seven African countries, including Ghana (baseline), were used in this study. As expected, MCB production has lower impacts (compared to Ghana) in DRC, Ethiopia, Zambia, and Kenya likely due to the higher percentage of renewable energy: ~ 99% in DRC and Ethiopia^65,66^;  ~ 88% in Kenya^67^; and ~ 86% in Zambia^68^. On the other hand, higher emission equivalents are observed for Mozambique and South Africa, potentially attributed to the greater consumption of fossil fuel energy^57^: Mozambique imports a substantial quantity of electricity from South Africa^69^ where ~ 80% of the total energy mix consists of coal^70,71^.

#### Alternatives processes and fuels

To minimise the overall impact of MCB production driven by electrical power usage, alternative models were created, excluding expendable equipment (e.g., incubators and grow tents, autoclave for sterilising tools). Because the drying process is essential, three alternative fuels that are common in Africa^72^ are proposed for fuelling the oven: liquified petroleum gas (LPG), firewood, and charcoal. Figure 3d shows the environmental impact of MCB production based on electricity (MCB-E), LPG (MCB-L), firewood (MCB-F), and charcoal (MCB-C); the data for these results are summarised in Table S6 in ‘Supplementary Material’. MCB-F shows a comparatively lower environmental impact, attributed to the overall carbon neutrality and the renewable nature of wood^61^, provided efficient filter fan units are installed. MCB-C production ranks second due to the higher impact of charcoal compared to wood: this can be attributed to the energy used in the process of converting wood to charcoal, along with the resulting emissions^73^. MCB-L exhibits a higher ecological footprint than MCB-F, MCB-C, and concrete bricks, especially for AP and POCP; this is attributed to the chemical composition of LPG and the emissions released during its combustion^74^.

### Cradle-to-grave LCA: comparison of MCB and concrete brick production

**Figure 4:**
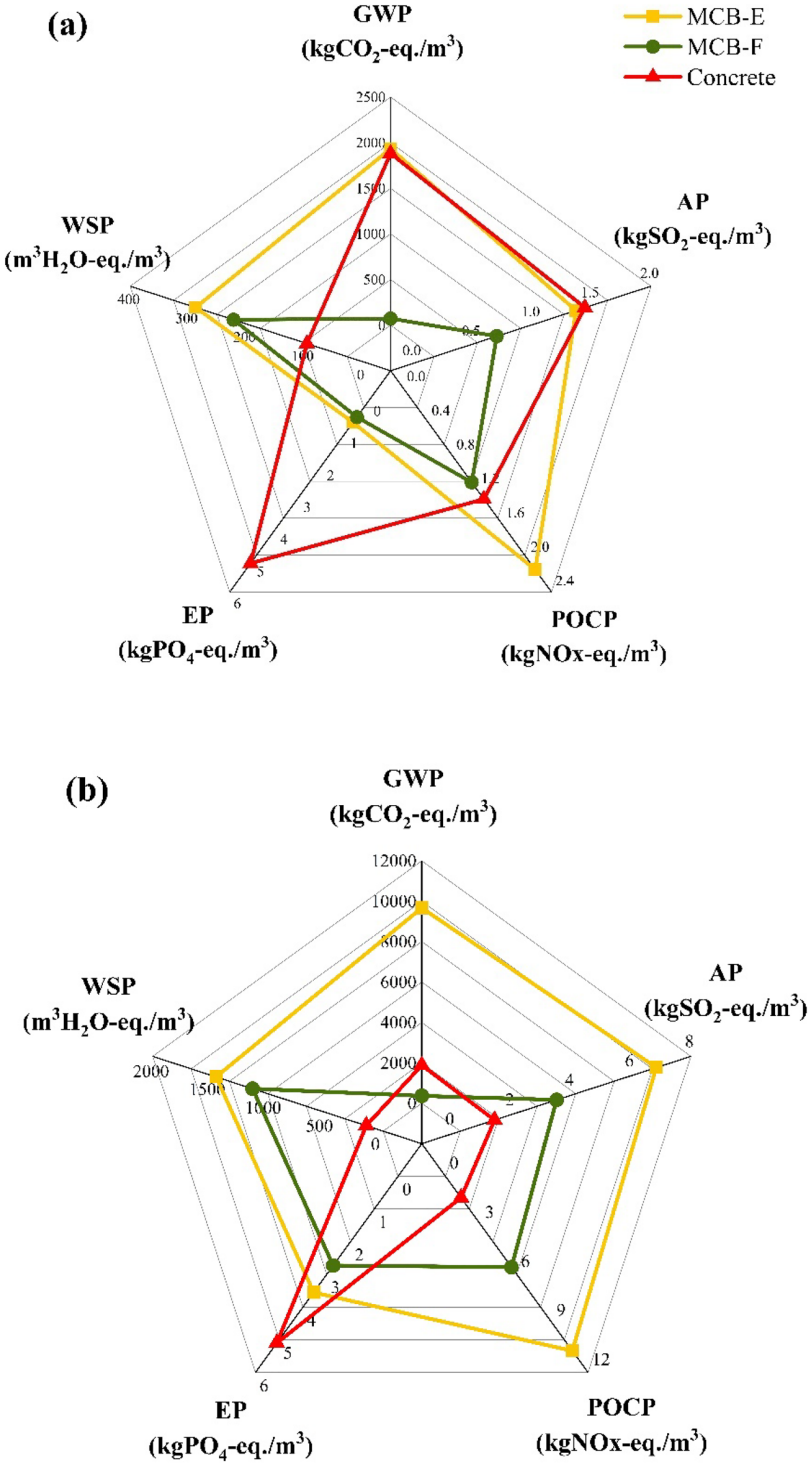
(**a**) 20-year and (**b**) 100-year cradle-to-grave impact of MCB-Es, MCB-Fs, and concrete bricks.

Figure 4 (data in Table S7) illustrates the complete life cycle environmental impact of MCBs based on electricity and firewood (representing the highest and lowest impact fuels for MCB, respectively), compared to concrete bricks. The figure shows the cradle-to-grave LCA results for two different time frames: 20 years (i.e., estimated lifespan for MC products)^54,55^ and 100 years (i.e., estimated lifespan for concrete buildings)^56^. The LCA graph for the 20-year time frame indicates that MCB-Fs have the lowest environmental impact, while the impact of MCB-Es is comparable to that of concrete bricks: the impact of MCB-Fs is ~ 50% lower than that of MCB-Es and ~ 5 times lower than that of concrete bricks. It is important to note that this is subject to the quality and composition of the wood as well as the efficiency of the emissions capture systems. In fact, uncontrolled combustion of wood can generate toxic emissions (*a.k.a*. flue gases) in the form of CO_2_, SO_2_, NOx, CH_4_, carbon monoxide (CO), nitrous oxide (N_2_O), ammonia (NH_2_), particulate matter (PM_10_), trace metals, dioxins, and furans among others^75^. If not controlled, the exposure to these flue gases can pose a threat to human health, causing severe cardiopulmonary, endocrine, and reproductive health concerns^76^.

The 100-year time frame graph in Fig. 4 shows that the impact of MCB-Fs is almost equivalent to that of concrete bricks, i.e., 103.1 Pt for MCB-Fs versus 104.5 Pt for concrete bricks on a single score impact scale. Meanwhile, the impact of MCB-Es is nearly double that of MCB-Fs and concrete bricks. It should also be acknowledged that the impact of concrete bricks appears to be lower than anticipated because, in many African countries, the method of production is less energy intensive than it is in Global-North countries. Furthermore, since the SimaPro software does not allow for temporal modelling, the 100-year life cycle for MCBs was modelled by multiplying the 20-year full life cycle by 5, thus, assuming that the total emissions over the 100-year life cycle are equivalent to 5 times the emissions for a 20-year life cycle. While these cradle-to-grave models assume that MCBs are landfilled after use (EOL), it is worth noting that MCBs are typically composted, a practice expected to significantly reduce their ecological footprint.

For a more comprehensive impact assessment, future LCA studies should take into account the CO_2_ emissions from the metabolic activity of the growing mycelium. While such studies are currently very limited, Livne et al.^16^ recently developed a method that estimates the metabolic CO_2_ production of specific fungal species as a function of the dry weight loss of the MCBs. It was found that, even though the metabolic CO_2_ increases the embodied carbon of a single MCB, the carbon footprint remains negative as the ratio of sequestered CO_2_ (by plants) to emitted CO_2_ (by fungi) is high. For example, the ratio for MCBs based on wheat straw and *Trametes betulina* fungus used by the authors was approximately 6:1, suggesting that MCBs can function as a CO_2_ sink compared to concrete bricks.

### Domestic and international hauls

**Figure 5:**
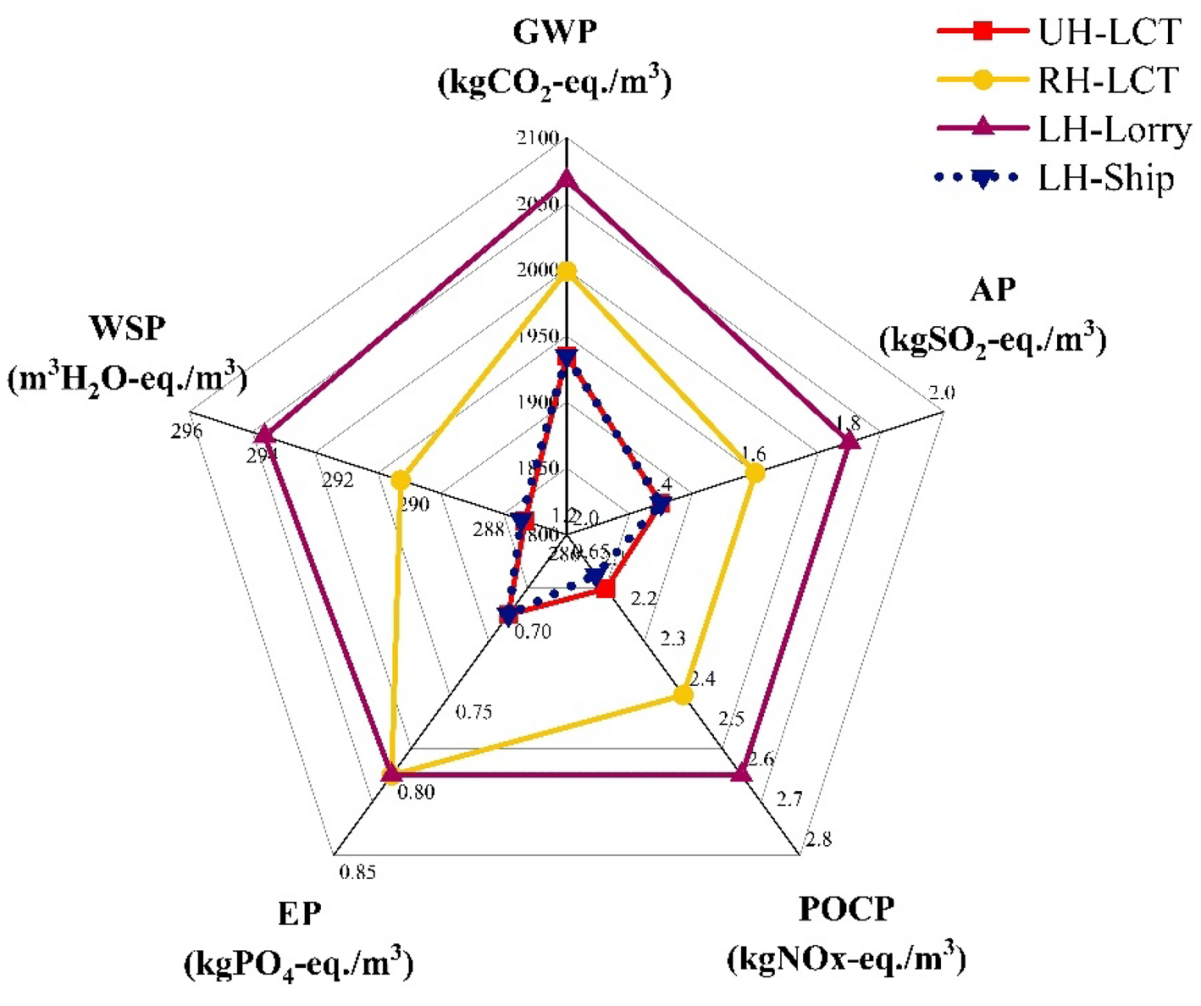
Environmental impact of MCBs based on product distribution scenarios.

While the emphasis of this study has been on the small-scale industrial production and local distribution of MCB products, it is also worth considering the potential for exportation from Ghana to foreign markets. Figure 5 depicts the potential footprint of MCBs resulting from the following distribution scenarios:

1. Local distribution using a light cargo truck (UH-LCV) over a travel distance of 3.5 km (i.e., assumption used for the MCB-E baseline study).
2. Domestic distribution over a travel distance of 500 km, e.g., from Kumasi (Ashanti region of Ghana) to Wa (Northern region); by road, using a light cargo truck (RH-LCV).
3. International export over a travel distance of 500 km, e.g., from Accra (Ghana) to Lagos (Nigeria); by road using a long-haul lorry (LH-Lorry).
4. International export over a travel distance of 500 km, e.g., from Accra to Lagos; by sea using a cargo ship (LH-Ship).

The travel distances for the long-haul models, i.e., RH-LCV, LH-Lorry, and LH-Ship, were maintained at 500 km for ease of comparison. The respective weights and cargo capacities of the proposed transport vessels vary significantly and have all been accounted for in the calculations. The chart (Fig. 5) suggests that employing a light cargo truck (UH-LCT) for local distribution results in a relatively low environmental impact, albeit one that is comparable to the environmental footprint associated with long-haul distribution by ship cargo (LH-Ship).Although LH-Lorry appears to have the greatest environmental impact, long-haul lorries are generally regarded as more fuel-efficient than light cargo trucks as they offer a higher payload capacity per cargo per distance travelled^77^. This is also valid for cargo ships, explaining the comparably lower impact of LH-Ship, although ships have a higher potential for ecosystem damage in the event of catastrophic accidents, e.g., oil spills^78^. Nonetheless, these results appear to support the hypothesis that local production and distribution of MCB products could be more sustainable than outsourcing.

## Recommendations

The findings from this LCA study point to (1) fuel source, (2) transport, (3) biomass composition, and (4) water usage as the key factors that influence the environmental impact of MCBs (and MCs more broadly). This section outlines recommendations to mitigate the damage contribution of these parameters, possibly reducing the ecological footprint of MCs.

### Fuel source

Conventional MC manufacturing methods involve the heavy use of electrically powered equipment, e.g., autoclaves, cleanrooms, incubators, ovens, and hot-presses. This results in electricity being the predominant factor contributing to the ecological footprint of this technology, with the culturing and post-processing stages being the major environmental hotspots (Fig. 2). As shown in Fig. 3c, the sensitivity to electricity seems to be notably higher in countries where fossil fuels are the primary energy sources (e.g., South Africa) and lower in those that mostly rely on renewable energy (e.g.*,* DRC and Ethiopia). Given that renewable energy alternatives (e.g., solar energy) have the potential to reduce the overall ecological footprint, individual stakeholders should be encouraged to use them as their primary source of energy. However, due to the high costs often associated with the installation and maintenance of these systems, MC producers may lean towards prioritising production costs, possibly at the expense of ecological soundness, by adopting cost-effective fuels. In Africa, traditional fuel alternatives like LPG, firewood, and charcoal are abundant, readily accessible, and overall cost-effective. The results from the sensitivity analysis (Fig. 3d) suggested that the use of LPG, a fossil-derived fuel, does not seem to significantly reduce the overall impact of MCB production even after eliminating expendable processes (e.g., autoclave sterilisation). Conversely, MCB production based on firewood and charcoal fuels showed notable reductions in the environmental footprint by ~ 70–80%.

In our recent review paper^10^, we emphasised the importance of integrating the MC technology with local practices to facilitate its establishment and potentially reduce production costs. However, we recognise that achieving complete integration might pose challenges for various reasons. For example, while capitalising on pre-established traditional clay ovens fuelled by firewood could offer a potential solution for drying MCs, it comes with the following limitations: restricted size for handling large production volumes; inefficient fuel combustion; lack of precise operational controls; and lack of filtration, capture, and treatment systems for flue emissions. Moreover, while there is anecdotal evidence indicating that firewood and charcoal are more economical than electricity for household use^63^, this may not necessarily hold true for MC production, especially in small- and large-scale industrial settings. Given that the drying process can extend up to 48 h for each batch, the quantities of wood and charcoal required per batch may surpass that of electricity or gas fuel, unless advanced fuel-efficient systems are employed. In view of this, forthcoming studies on the sustainability of MCs in Africa should consider the financial implications of MC production based on different fuel sources using tools such as Life Cycle Costing (LCC) and Cost–Benefit analyses.

### Transport

The environmental impact of MCs is further influenced by transportation in two ways: distance travelled (Fig. 3b) and mode of transport (Fig. 5). There appears to be a linear correlation between the travel distance and the resulting footprint of MCs, suggesting that local sourcing could be more ecologically beneficial and should be prioritised over outsourcing. Efficient strategies to reduce emissions generated during material sourcing and product distribution, e.g., employing eco-friendly and fuel-efficient vehicles and optimising transport routes, are also crucial considerations. For example, lorries could be more suitable for long-haul journeys, as suggested by the LCA results (Fig. 5), due to the higher payload capacity per cargo over distance travelled, and overall fuel efficiency^77^. Cargo ships (where possible) also present a viable solution for long distance domestic or international distribution^78^.

It is also important to consider the fuel emissions of the vehicles used. It is imperative that MC manufacturers and distributors comply with road emission standards by investing in low emission cargos and attending periodic mandatory roadworthiness tests to monitor the state of their vehicles. Unfortunately, only a few African nations have and enforce emissions standards, as mentioned in a recent study by Ayetor et al.^79^ Based on the findings of their study, it could also be inferred that the environmental impact contribution from transportation of MCs is likely to be higher in countries such as Nigeria, South Africa, Egypt, Morocco, Algeria, Libya, and Ethiopia. In fact, the authors noted that cumulatively, these seven countries alone are responsible for ~ 70% of the total emissions from the transport sector in Africa, stemming from the use of low-quality fuels, the prevalence of older vehicles, and the lack of mandatory road worthy laws. This emphasises the need for stakeholders involved in the MC industry, particularly in the seven listed countries, to earnestly embrace sustainable practices. Such dedication is crucial, not just for fostering the environmental sustainability of MC technology in Africa but also to actively contribute to the continent’s overarching goal of reducing its carbon footprint^80,81^.

### Biomass composition

The findings of this study (Section "Electricity, water, and biomass consumption") demonstrate that biomass composition has the potential to significantly influence the sustainability of MCs; the results also suggest that substrate mixes with higher concentrations of nutrient-rich biomass may possibly have a higher damage contribution to the overall environmental impact (Fig. 2). Even though this study was modelled on the assumption that maize grains only constitute 20 wt% of the total MC substrate, the results indicate a higher potential for acidification, photochemical ozone creation, eutrophication, and water scarcity compared to the bulk substrate (i.e., coconut coir). This may be directly related to the higher water and nutrient (supplemented by fertilisers) requirements of nutrient-rich biomass as compared to biomass with lower nutritional values. It is also worth noting that the EcoInvent 3 dataset for maize grains does not consider the possibility that this biomass could be sourced from post-harvest and post-processing waste, in which case the environmental footprint could be lower. Future studies should also explore the viability of incorporating expired and slightly deteriorated biomass (from post-harvest, post-processing, storage, and post-consumer operations), especially those based on glucose and starch, as biomass or nutrient supplements for fungal growth. This would not only add value to materials otherwise considered waste; it could also potentially expedite mycelium growth and enhance its density.

Another key factor that can influence the choice of biomass for MCs is agricultural waste availability. It is well-known that the African continent is characterised by a diverse array of climates, with some countries even featuring multiple climates within their borders (e.g., South Africa). This climate diversity and the resulting seasonal variations play a pivotal role in determining the types of crops that are accessible and, consequently, the composition of the biomass reserve. This may present a problem when specific biomass compositions are required to achieve consistent MC properties and, during seasonal scarcity, manufacturers may be compelled to outsource materials. In other cases, the required agricultural biomass may be only available in areas that are not geographically or financially optimal for outsourcing or establishing a production facility. If not accounted for, these scenarios could prompt manufacturers to seek alternatives that may worsen the environmental impact of MCs related to the use of organic biomass. For instance, they might encourage local farmers (e.g., through financial incentives) to resort to genetic modification and increase the doses of fertilisers for higher crop yields.

Additional factors influencing the choice of biomass composition will depend on the specific needs of the selected fungal species, the potential for the biomass to generate toxic emissions upon fungal degradation, and various ecological, social, and economic considerations.

### Water consumption

The substantial water requirements for hydrating biomass to support fungal growth coupled with water used in operating sterilisation systems increase the possibility of water scarcity and deprivation (Fig. 2). The model proposed in this study assumes that water is 100% lost as wastewater that is discharged into the environment. However, hydration water stored in the MC would likely be lost as water vapour in the drying process, while water used to operate the autoclave can be used over an extended period for multiple production batches. Notwithstanding, water in vapour form and contaminated water could be partially reclaimed by implementing efficient water capture and treatment systems. This approach also has the potential to alleviate the risk of eutrophication associated with the discharge of wastewater contaminated with organic biomass.

As the water used throughout the production process undergoes complete sterilisation, the use of contaminated wastewater should not markedly affect fungal growth. Moreover, fungal organisms are known for their ability to decompose toxic heavy metals, microplastics, and microbes^82–84^. While these assertions require validation through research, the possibility of repurposing contaminated water could present opportunities for utilising wastewater from other industrial processes and from harvested rainwater. The impact associated with water consumption is also affected by water availability, which is directly related to climate characteristics and seasonal variations. Tropical areas usually boast ample water reserves, particularly during the rainy season, that is partly used for irrigation, household consumption, or stored for use in the dry season^85^. In these regions, rainwater could be collected, stored, and utilised to produce MCBs. In contrast, water is scarce in arid (desert) regions and during the dry seasons, presenting challenges for the operation of an MC production chain.

### Land use

It is important to use agricultural resources carefully when manufacturing biobased alternatives to prevent excessive damage to the land. The environmental impact caused by the use and transformation of land for human activities can be quantified using the land use impact metric. Land use evaluates both the direct damages of these activities (e.g., agriculture) on terrestrial ecosystems and their broader, indirect effects on the environment^86,87^. According to the FAO, agricultural land use emissions account for up to 24% of the global GHG emissions, making them significant contributors to climate change^88^. This is mainly due to the release of stored CO_2_ caused by deforestation, drainage of peatlands, grassland transformation, and other land conversion processes. Moreover, such processes reduce bioresource availability and alter the landscape of terrestrial habitats, potentially leading to biodiversity loss and destabilising the energy dynamics of the ecosystems^87^.

While this work does not address the land use impact category, insights can be obtained from the ReCiPe midpoint damage charts illustrated in Figs. S1 and S2 (Supplementary Materials). As expected, the charts suggest that biomass sourcing is the largest contributor to land use damage. Furthermore, a notable difference can be observed between the impacts of maize and coconut coir, respectively, which might be attributed to several factors. The primary reason for the impact difference could be the fact that the EcoInvent database treats maize as a crop primarily cultivated for MCB production, and not as biomass obtained from agricultural waste and byproducts which would be otherwise discarded. The disparity may also be influenced by the distinct cultivation processes and practices^89^. Annual crops like maize complete their lifecycle within a single growing season and, therefore, are planted, harvested, and replanted every year. The need for extensive soil preparation (e.g., ploughing) before each season can alter the composition and characteristics of the soil^90^. On the other hand, coconut is cultivated on perennial tree crops that also provide a stable soil environment for soil organisms and contribute to carbon sequestration^91^.

## Concluding remarks

The goal of this study was to estimate the ecological footprint of MCBs in the context of African countries and compare it with that of conventional construction materials like concrete bricks, using an LCA methodology. As expected, the impact of MCB production is notably affected by the electrical power commonly used for autoclaves, cleanrooms, incubators, and ovens; thus, making the culture and post-processing phases the major environmental hotspots of production. This is further exacerbated in countries like South Africa, where electricity is predominantly generated from coal. However, by opting for more sustainable fuel alternatives like firewood (with efficient filtration systems) and optimising the MCB production process, the overall footprint of MCBs can be reduced by up to 50%. These solutions may also prove to be more financially viable, especially in many rural communities in Africa where electricity can be unaffordable or unreliable. The findings of this study also suggest that the impact of MCBs can be further mitigated by managing water usage, biomass sourcing and composition, as well as travel distance and mode of transportation.

The shorter lifespan and lower strength of MCBs can also have an influence on their sustainability and restrict their use. For instance, if MCBs are used for a wall that is expected to last approximately 100 years, each MCB would need replacement at the end of its 20-year lifecycle until the wall's intended lifespan is reached. On the other hand, due to the low strength of MCBs, using multiple MCBs may be necessary to meet the strength requirement that could be achieved with a single traditional masonry brick.

We acknowledge the shortcomings of our simplified model and recommend that future LCAs should provide a more comprehensive analysis, considering mechanical property requirements as well as the health, social, and economic costs associated with MCB production in Africa. Nevertheless, the finding of this study provide insights that can be used to proactively identify and address potential environmental repercussions of the MC technology in Africa.

### Supplementary Information

Supplementary Information.

## Data Availability

Data used, generated and/or analyzed in this study can be found on the University of Bristol’s repository https://data.bris.ac.uk/data/.
