# Supplementary material for: Potential environmental impact of mycelium composites on African communities
Source: Sci Rep. 2024 May 24;14:11867. doi: 10.1038/s41598-024-62561-7 (PMC11126690; doi:10.1038/s41598-024-62561-7)
Supplement: Supplementary file 1 — Supplementary Information. [file 41598_2024_62561_MOESM1_ESM.docx]

# Supplementary Information

# Potential Environmental Impact of Mycelium Composites on African Communities

*Stefania Akromah*, Neha Chandarana*, Jemma L. Rowlandson*, Stephen J. Eichhorn**

Bristol Composites Institute, School of Civil, Aerospace, and Design Engineering, Faculty of Science and Engineering, University of Bristol, University Walk, Bristol, BS8 1TR, UK

E-mails: s.akromah@bristol.ac.uk; neha.chandarana@bristol.ac.uk;
j.rowlandson@bristol.ac.uk; s.j.eichhorn@bristol.ac.uk

ORCID identification numbers: 0000-0002-4578-2419; 0000-0002-6361-1533; 0000-0001-8450-1096; 0000-0003-4101-273X

**Table S1** Environmental impact of MCB production (per cubic meter), showing the contribution of the input parameters.

| **Inputs** | **kgCO_2_-eq.** | **kgSO_2_-eq.** | **kgNOx-eq.** | **kgPO_4_-eq.** | **m^3^H_2_O-eq.** | **Single Score Damage**  **(Pt)** |
| --- | --- | --- | --- | --- | --- | --- |
| Electricity consumption | 1149.890 | 0.794 | 1.223 | 0.206 | 71.899 | 24.0 |
| Water consumption | 4.408 | 0.019 | 0.012 | 0.009 | 182.585 | 0.49 |
| Transport | 7.415 | 0.032 | 0.039 | 0.008 | 0.568 | 0.33 |
| Nutrient biomass | -62.579 | 0.317 | 0.608 | 0.153 | 187.181 | 1.79 |
| Substrate biomass | 0.177 | 0.001 | 0.001 | 0.00 | 0.010 | 0.01 |
| Acrylic varnish | 0.080 | 0.001 | 0.00 | 0.00 | 0.052 | 0.04x10^-1^ |
| PP grow bags | 0.251 | 0.001 | 0.001 | 0.00 | 0.103 | 0.01 |
| Disposable gloves | 1.346 | 0.004 | 0.002 | 0.00 | 0.711 | 0.04 |
| Isopropanol | 2.082 | 0.008 | 0.005 | 0.002 | 0.556 | 0.08 |
| Waste biomass | 22.796 | 0.002 | 0.00 | 0.111 | 0 | 0.47 |
| Wastewater | 3.174 | 0.019 | 0.021 | 0.057 | -162.530 | 0.05 |
| Waste PP grow bags | 0.012 | 0.00 | 0.00 | 0.001 | 0.001 | 0.01x10^-1^ |
| Waste disposable gloves | 0.029 | 0.00 | 0.00 | 0.001 | 0.003 | 0.01 |
| **Total** | **1,129.080** | **1.197** | **1.911** | **0.549** | **281.140** | **27.29** |

**Table S2** Environmental impact (equivalent emissions, midpoint damage, and single score endpoint damage) of the production process for one cubic meter of MCBs. (HH = human health; TE = terrestrial ecosystems; FE = freshwater ecosystems; AE = aquatic ecosystems; DALY = Disability Adjusted Life Years; USD2013 = United States Dollars in the year 2013)

| **Impact Category** | **Unit** | **Material Prep.** | **Sterilisation** | **Incubation** | **Post-Process** | **Waste** |
| --- | --- | --- | --- | --- | --- | --- |
| Global Warming Potential | kgCO_2_-eq | -53.57 | 85.38 | 441.69 | 629.67 | 26.01 |
| Acidification Potential | kgSO_2_-eq. | 0.35 | 0.08 | 0.31 | 0.44 | 0.02 |
| Photochemical Ozone Creation Potential | kgNOx-eq. | 0.65 | 0.10 | 0.47 | 0.67 | 0.02 |
| Eutrophication Potential | kgPO_4_-eq. | 0.16 | 0.02 | 0.08 | 0.11 | 0.17 |
| Water Deprivation Potential | m^3^-eq. | 197.19 | 178.82 | 28.06 | 39.42 | -162.53 |
| **Midpoint Damage** | | | | | | |
| Global Warming (HH) | DALY | 4.26x10^-5^ | 8.00x10^-5^ | 4.14x10^-4^ | 5.90 x10^-4^ | 2.34 x10^-5^ |
| Global Warming (TE) | species.yr | 1.29 x10^-7^ | 2.42 x10^-7^ | 1.25 x10^-6^ | 1.78 x10^-6^ | 7.06 x10^-8^ |
| Global Warming (FE) | species.yr | 3.51 x10^-12^ | 6.60 x10^-12^ | 3.41 x10^-11^ | 4.87 x10^-11^ | 1.93 x10^-12^ |
| Stratospheric Ozone Depl. | DALY | 2.38 x10^-7^ | 2.13 x10^-8^ | 1.05 x10^-7^ | 1.50 x10^-7^ | 3.23 x10^-9^ |
| Ionizing Radiation | DALY | 7.86 x10^-9^ | 5.66 x10^-9^ | 6.35 x10^-9^ | 8.77 x10^-9^ | 8.61 x10^-10^ |
| POCP (HH) | DALY | 1.62 x10^-7^ | 7.61 x10^-8^ | 3.68 x10^-7^ | 5.20 x10^-7^ | 8.04 x10^-9^ |
| Fine Particulates | DALY | 5.77 x10^-5^ | 1.65 x10^-5^ | 5.86 x10^-5^ | 8.10 x10^-5^ | 4.56 x10^-6^ |
| POCP (TE) | species.yr | 2.34 x10^-8^ | 1.10 x10^-8^ | 5.34 x10^-8^ | 7.53 x10^-8^ | 1.16 x10^-9^ |
| Terrestrial Acidification | species.yr | 7.38 x10^-8^ | 1.22 x10^-8^ | 5.11 x10^-8^ | 7.05 x10^-8^ | 3.88 x10^-9^ |
| Freshwater Eutrophication | species.yr | 1.26 x10^-8^ | 3.12 x10^-9^ | 7.67 x10^-9^ | 9.99 x10^-9^ | 8.21 x10^-8^ |
| Marine Eutrophication | species.yr | 6.50 x10^-11^ | 6.06 x10^-13^ | 1.33 x10^-12^ | 1.78 x10^-12^ | 1.01 x10^-10^ |
| Terrestrial Ecotoxicity | species.yr | 2.26 x10^-9^ | 8.44 x10^-10^ | 3.86 x10^-9^ | 5.38 x10^-9^ | 1.54 x10^-10^ |
| Freshwater Ecotoxicity | species.yr | 1.45 x10^-9^ | 1.12 x10^-9^ | 5.37 x10^-9^ | 7.63 x10^-9^ | 5.07 x10^-10^ |
| Marine Ecotoxicity | species.yr | 2.52 x10^-10^ | 2.14 x10^-10^ | 1.02 x10^-9^ | 1.45 x10^-9^ | 1.05 x10^-10^ |
| Human Carcinogenic Tox. | DALY | 7.81 x10^-6^ | 1.03 x10^-5^ | 1.84 x10^-5^ | 2.59 x10^-5^ | 2.80 x10^-6^ |
| Human Non-Carcinogenic Tox. | DALY | 9.83 x10^-6^ | 3.46 x10^-6^ | 1.35 x10^-5^ | 1.88 x10^-5^ | 7.82 x10^-6^ |
| Land Use | species.yr | 2.01 x10^-7^ | 6.25 x10^-9^ | 2.96 x10^-8^ | 4.23 x10^-8^ | 1.82 x10^-9^ |
| Mineral Resource Scarcity | USD2013 | 0.05 | 0.02 | 0.08 | 0.11 | 0.01 |
| Fossil Resource Scarcity | USD2013 | 3.49 | 12.42 | 66.20 | 94.08 | 0.14 |
| Water consumption (HH) | DALY | 3.32x10^-6^ | 8.71 x10^-6^ | -1.14 x10^-6^ | -1.73 x10^-6^ | -8.41 x10^-6^ |
| Water consumption (TE) | species.yr | 2.04 x10^-8^ | 5.76 x10^-8^ | 1.77 x10^-8^ | 2.48 x10^-8^ | -5.11 x10^-8^ |
| Water consumption (AE) | species.yr | 1.76 x10^-11^ | 3.08 x10^-12^ | 3.34 x10^-12^ | 4.76 x10^-12^ | -2.27 x10^-12^ |
| **Endpoint Damage** | | | | | | |
| Single Score (Total) | Pt | 2.19 | 2.17 | 9.26 | 13.14 | 0.53 |

**Table S3** Sensitivity of equivalent emissions from the production of one cubic meter of MCBs to electricity, water, and organic biomass.

| **Sensitivity Parameter** | **Impact** | **Low** | **Baseline** | **High** | **% Variation** |
| --- | --- | --- | --- | --- | --- |
| **Electricity** | kgCO_2_-eq. | -20.819 | 1129.08 | 2278.97 | ±101.8% |
|  | kgSO_2_-eq. | 0.424 | 1.232 | 2.04 | ±65.6% |
|  | kgNOx-eq. | 0.688 | 1.911 | 3.134 | ±64.0% |
|  | kgPO_4_-eq. | 0.343 | 0.549 | 0.755 | ±37.5% |
|  | m^3^-eq. | 209.241 | 281.140 | 353.039 | ±25.6% |
| **Water** | kgCO_2_-eq. | 1121.498 | 1129.08 | 1136.663 | ±0.7% |
|  | kgSO_2_-eq. | 1.192 | 1.232 | 1.271 | ±3.2% |
|  | kgNOx-eq. | 1.878 | 1.911 | 1.945 | ±1.8% |
|  | kgPO_4_-eq. | 0.483 | 0.549 | 0.615 | ±12.0% |
|  | m^3^-eq. | 261.085 | 281.140 | 301.194 | ±7.1% |
| **Biomass** | kgCO2-eq. | 1168.686 | 1129.08 | 1089.474 | ±3.5% |
|  | kgSO2-eq. | 0.895 | 1.232 | 1.569 | ±27.4% |
|  | kgNOx-eq. | 1.304 | 1.911 | 2.519 | ±31.8% |
|  | kgPO_4_-eq. | 0.285 | 0.549 | 0.813 | ±48.1% |
|  | m^3^-eq. | 93.948 | 281.140 | 468.332 | ±66.6% |

**Table S4** Sensitivity of equivalent emissions (per cubic meter) from MCB production to travel distance. (KSI = Kumasi; SUN = Sunyani; ACC = Accra; WA = Wa).

| **Travel Distance** | **kgCO_2_-eq.** | **kgSO_2_-eq.** | **kgNOx-eq.** | **kgPO_4_-eq.** | **m^3^H_2_O-eq.** | **Single Score Damage**  **(Pt)** |
| --- | --- | --- | --- | --- | --- | --- |
| **Baseline (3.5 km)** | 1,129.08 | 1.232 | 1.911 | 0.549 | 281.14 | 27.29 |
| **KSI – SUN (125 km)** | 1,386.2 | 2.380 | 3.264 | 0.833 | 300.843 | 38.89 |
| **KSI – ACC (250 km)** | 1,650.74 | 3.562 | 4.656 | 1.125 | 321.114 | 50.82 |
| **KSI – WA (500 km)** | 2,179.82 | 5.926 | 7.439 | 1.710 | 361.656 | 74.70 |
| **WA – ACC (800 km)** | 2,814.71 | 8.762 | 10.779 | 2.412 | 410.306 | 103.34 |
| **Emission/km (Slope)** | 2.1163 | 0.0095 | 0.0111 | 0.0023 | 0.1622 | 0.0955 |

**Table S5** Sensitivity of equivalent emissions (per cubic meter) from MCB production to energy mix.

| **Country** | **kgCO_2_-eq.** | **kgSO_2_-eq.** | **kgNOx-eq** | **kgPO4 eq.** | **m^3^H_2_O-eq.** | **Single Score Damage**  **(Pt)** |
| --- | --- | --- | --- | --- | --- | --- |
| Ghana | 1129.08 | 1.23 | 1.91 | 0.55 | 281.14 | 27.29 |
| Ethiopia | 22.61 | 0.60 | 0.80 | 0.40 | 218.58 | 5.83 |
| DR Congo | 32.61 | 0.69 | 0.85 | 0.40 | 218.02 | 6.12 |
| Zambia | 543.52 | 5.43 | 2.93 | 1.42 | 247.76 | 32.18 |
| Kenya | 704.37 | 6.06 | 3.51 | 0.80 | 249.93 | 34.08 |
| Mozambique | 1679.19 | 18.23 | 9.04 | 4.58 | 410.22 | 90.99 |
| South Africa | 3564.36 | 43.64 | 20.50 | 10.59 | 514.05 | 204.68 |

**Table S6** Sensitivity of equivalent emissions (per cubic meter) from MCB production to fuel source, compared to concrete brick production. (E = electricity; L = liquified petroleum gas; F = firewood; C = charcoal).

| **Process** | **kgCO_2_-eq.** | **kgSO_2_-eq.** | **kgNOx-eq.** | **kgPO_4_-eq.** | **m^3^H_2_O-eq.** | **Single Score Damage**  **(Pt)** |
| --- | --- | --- | --- | --- | --- | --- |
| **MCB-E** | 1129.08 | 1.23 | 1.91 | 0.55 | 281.14 | 27.29 |
| **MCB-L** | 294.87 | 3.22 | 1.93 | 0.65 | 223.1 | 19.34 |
| **MCB-F** | -732.35 | 0.57 | 0.89 | 0.38 | 215.96 | 5.92 |
| **MCB-C** | 47.08 | 0.59 | 0.87 | 0.39 | 290.52 | 8.08 |
| **Concrete** | 346.48 | 0.85 | 1.04 | 0.25 | 72.64 | 9.94 |

**Table S7** Environmental impact per cubic meter of MCBs compared to concrete bricks over 20- and 100-year timeframes. (E = electricity; L = liquified petroleum gas; F = firewood; C = charcoal).

| **Brick Type** | **kgCO_2_-eq.** | **kgSO_2_-eq.** | **kgNOx-eq.** | **kgPO_4_-eq.** | **m^3^H^2^O-eq.** | **Single Score Damage**  **(Pt)** |
| --- | --- | --- | --- | --- | --- | --- |
| ***20 years*** | | | | | | |
| MCB-E | 1934.81 | 1.37 | 2.14 | 0.71 | 287.08 | 41.98 |
| MCB-F | 73.38 | 0.70 | 1.11 | 0.55 | 221.90 | 20.61 |
| Concrete | 1881.87 | 1.44 | 1.31 | 5.10 | 95.32 | 104.48 |
| ***100 years*** | | | | | | |
| MCB-E | 9674.06 | 6.85 | 10.68 | 3.55 | 1435.40 | 209.88 |
| MCB-F | 366.90 | 3.52 | 5.56 | 2.73 | 1109.52 | 103.07 |
| Concrete | 1881.87 | 1.44 | 1.31 | 5.10 | 95.32 | 104.48 |


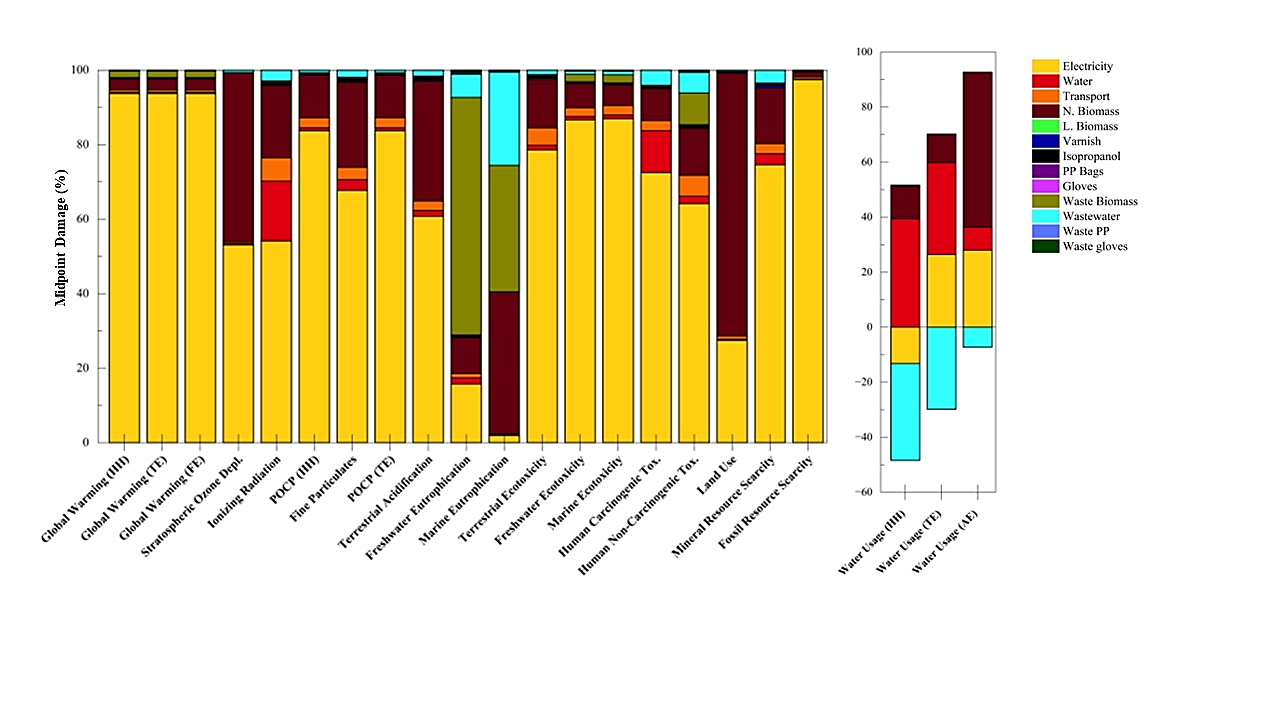


**Figure S1** Environmental impact of MCB production (midpoint damage) showing the contribution of the input parameters.


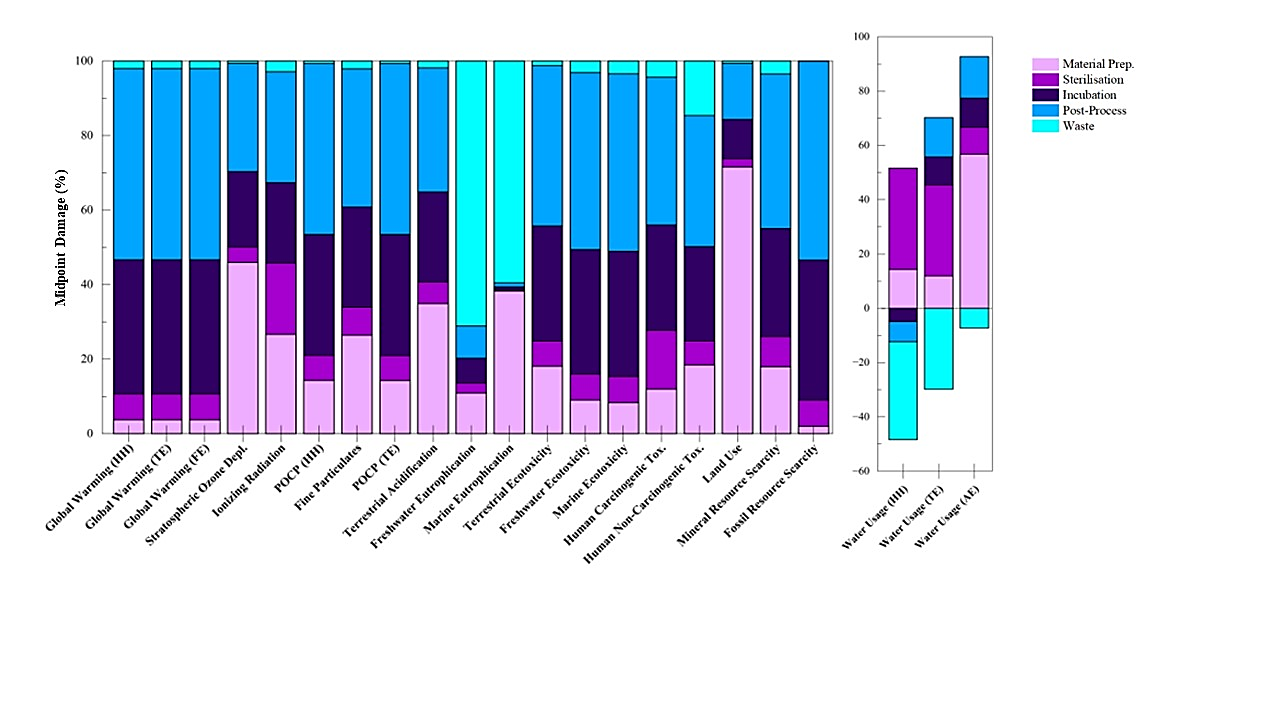


**Figure S2** Environmental impact (midpoint damage) of MCB production showing the impact contribution of the process stages.





**(b)**

**(a)**

**Figure S3** Environmental impact (endpoint damage) of MCB production showing the (a) impact contribution of the process stages and (b) environmental hotspots.
